# Breast cancer detection with upstream data fusion, machine learning, and automated registration: initial results

**DOI:** 10.1117/1.JMI.10.S2.S22409

**Published:** 2023-06-06

**Authors:** Lisa A. Mullen, William C. Walton, Michael P. Williams, Keith S. Peyton, David W. Porter

**Affiliations:** a Johns Hopkins Medicine, Breast Imaging Division, Baltimore, Maryland, United States; b Johns Hopkins University Applied Physics Laboratory, Laurel, Maryland, United States; c Independent Consultant, Annandale, Virginia, United States

**Keywords:** artificial intelligence, fusion, mammography, ultrasound, breast cancer, neural network registration

## Abstract

**Purpose:**

To develop an artificial intelligence algorithm for the detection of breast cancer by combining upstream data fusion (UDF), machine learning (ML), and automated registration, using digital breast tomosynthesis (DBT) and breast ultrasound (US).

**Approach:**

Our retrospective study included examinations from 875 women obtained between April 2013 and January 2019. Included patients had a DBT mammogram, breast US, and biopsy proven breast lesion. Images were annotated by a breast imaging radiologist. An AI algorithm was developed based on ML for image candidate detections and UDF for fused detections. After exclusions, images from 150 patients were evaluated. Ninety-five cases were used for training and validation of ML. Fifty-five cases were included in the UDF test set. UDF performance was evaluated with a free-response receiver operating characteristic (FROC) curve.

**Results:**

Forty percent of cases evaluated with UDF (22/55) yielded true ML detections in all three images (craniocaudal DBT, mediolateral oblique DBT, and US). Of these, 20/22 (90.9%) produced a UDF fused detection that contained and classified the lesion correctly. FROC analysis for these cases showed 90% sensitivity at 0.3 false positives per case. In contrast, ML yielded an average of 8.0 false alarms per case.

**Conclusions:**

An AI algorithm combining UDF, ML, and automated registration was developed and applied to test cases, showing that UDF can yield fused detections and decrease false alarms when applied to breast cancer detection. Improvement of ML detection is needed to realize the full benefit of UDF.

## 1 Introduction

Breast cancer is the most common cancer worldwide, and a leading cause of cancer death for women. In 2020 alone, more than 2.3 million women were diagnosed with breast cancer and 685,000 women died due to the disease globally.^1^^–^^3^ Early detection of breast cancer is known to dramatically decrease the morbidity and mortality of breast cancer, with mortality rates decreased between 20% and 40%, depending on the age and frequency of screening mammography.^4^

Screening examinations have used two-dimensional full field digital mammography (FFDM), which consists of two images of each breast, and have demonstrated sensitivity and specificity for detection of breast cancer as high as 89% and 72%, respectively.^5^ The development of digital breast tomosynthesis (DBT) has improved sensitivity and specificity to 90% and 79%, respectively.^5^ With DBT technology, multiple projections of each breast are obtained and then reconstructed into 1 mm slices. A major limitation of mammography is overlap of normal breast tissue, which obscures lesions. DBT addresses this issue by decreasing superimposition of normal tissue, thereby improving lesion detection and increasing accuracy.^6^ Despite the well-documented benefits of breast cancer screening and improved performance with the use of DBT, there are limitations. Mammography, FFDM and DBT, has decreased sensitivity in patients with dense breast tissue, as dense tissue can overlap and obscure lesions.^7^ Breast radiologists can be overwhelmed by the high volume of studies that are a necessary consequence of widespread screening programs, a problem made worse by DBT, with 200 to 400 images per study, compared to only 4 images per FFDM study. The task of interpreting mammography is difficult, and there is significant inter- and intra-reader variability.^8^

Breast ultrasound (US) can be used as a supplemental study to improve detection of breast cancers in dense breast tissue. Unfortunately, US also detects many false-positive lesions. Although it is well known that US detects additional cancers when paired with mammography, low specificity is a challenge.^9^^–^^11^

Due to the significant challenge of interpretation of mammography and breast US, and the ever-expanding volume of images, recent research has focused on the development of artificial intelligence algorithms for breast cancer detection.^12^^–^^14^ The potential benefit of artificial intelligence is the reduction of radiologist interpretation time without compromising performance.^15^^,^^16^

Upstream data fusion (UDF) is a unique concept that was developed to assist military operators in detection of military targets and analysis of images of potential targets.^17^^,^^18^ UDF can also be used to analyze medical images, in combination with machine learning (ML) and automated registration. ML is used to obtain candidate detections of lesions from DBT and US images with intentionally low thresholds to keep detection rates high at the expense of false positives. The false positives are reduced later by UDF processing. UDF performs data association of detections originating from the same lesions to match lesions in the images and provide probabilities of malignancy for fused detections.

Automated registration uses ML methods to construct deformation fields for the breast in different positions to map corresponding locations between images. Then UDF can match detected lesions with similar locations. But UDF does more than just match lesions with similar locations. It also matches lesions of similar cancer-likeness determined by ML models from lesion features. Location information and feature information are mathematically expressed in terms of statistical likelihood functions so statistical inference can be used to produce fused detection reports. By combining information across multiple images, the false alarms in the candidate detections from individual images are reduced. Fused reports are output for malignant lesions, lesion locations, and association of data in images from the same lesions. Also statistical confidence and uncertainty measures can be provided for the reports.

The purpose of our project was to develop an artificial intelligence algorithm by combining UDF, ML, and automated registration for the detection of breast cancer using both DBT and US.

## 2 Materials and Methods

### 2.1 Study Design Overview

The UDF system^17^ was evaluated as part of a retrospective study. The objective of the study was to apply UDF and ML to jointly process multiple images of the same lesion, including different modalities (DBT and US) and different projections [craniocaudal (CC) and mediolateral oblique (MLO)], to improve lesion detection, localization, and classification. This is in contrast to traditional CAD techniques, which process images from a single modality or without regard to viewing angle. Our study focused on the joint processing of the DBT CC view, DBT MLO view, and US images taken of the same lesion(s), thus, applying UDF to three image products simultaneously.

#### 2.2 Data

The primary dataset used for the project was a set of DBT and US data from Johns Hopkins Medicine (JHM). A second dataset, which was used for part of the system, involved a publicly available DBT data set from the Duke University Health System, which is available online at The Cancer Imaging Archive (TCIA) site.^19^^,^^20^ Both the JHM and Duke University data sets were approved by the respective institutional review boards with a waiver of informed consent; hence, the use of the datasets for our study is compliant with the Health Insurance Portability and Accountability Act.

First, we provide details on the selection criteria and curation of the JHM DBT and US data for our study. Cases for the study were selected based on the following inclusion criteria.

(1) Images were obtained between April 2013 and January 2019.
(2) Candidate patient studies must contain a biopsy-proven breast lesion.
(3) Studies must contain DBT-CC, DBT-MLO, and US images for candidate lesions.
(4) DBT and US images must have been taken within 3 months of each other.
(5) Image data must be provided in Digital Imaging and Communications in Medicine (DICOM) format.
(6) Details on the clinical diagnosis (i.e., clinical radiology report) must be available for each modality.
(7) Data must be deidentified.

Subsequent analysis of data and processing steps resulted in additional constraints on the data selection. Specifically, it was decided to include only patient studies containing mass lesions, and then only studies with single masses. Other abnormality types, such as calcifications, asymmetries, and architectural distortions, as well as studies with multiple lesions, were left for future research. The selection of only one, or a subset, of abnormality types is common in breast cancer ML research. For instance, the TCIA DBT dataset also excludes calcifications.^19^

**Fig. 1:**
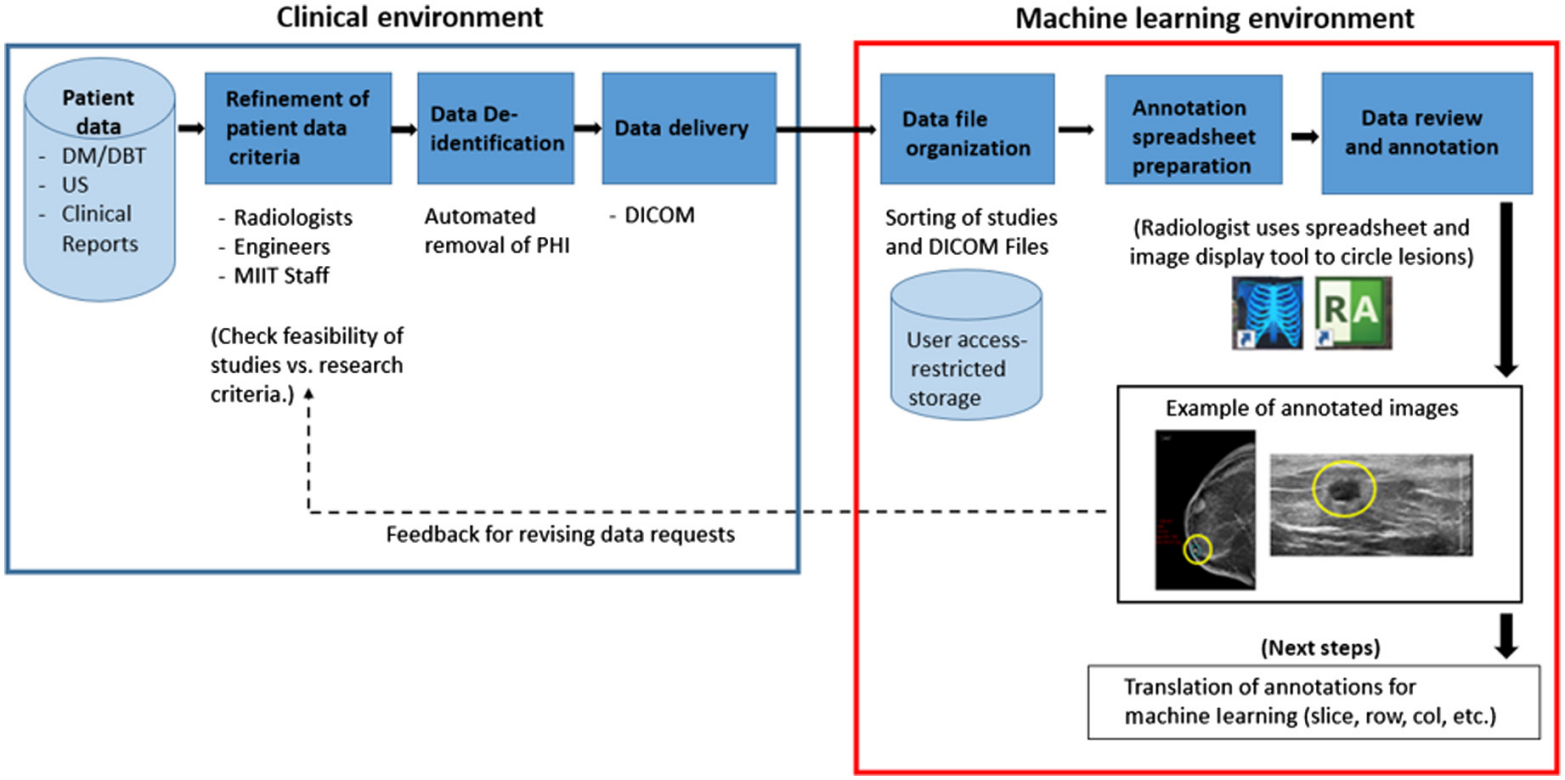
Data curation pipeline.

The overall sequence of steps taken to collect and curate data for experimentation is shown in Fig. 1. First, clinical data that met the desired requirements were deidentified and then transferred, in DICOM format, to a separate ML research facility. Next, after file storage and organization, an extensive data annotation effort was conducted in which one senior breast imaging radiologist manually circled the lesion on a single image from each of the three image types (CC, MLO, and US) for a given lesion. Image coordinate and size information for the annotations [i.e., regions-of-interest), along with lesion diagnosis (e.g., malignant versus benign), and other information were captured in a tabular (i.e., spreadsheet) format for supporting ML processing.

Table 1 shows data counts for the studies. A total of 875 studies were collected for which there existed a CC, MLO, and US image set with corresponding clinical diagnosis information. Of these, 556 were annotated during the timeframe of the study, given available resources. However, among these, only 150 were tabulated for experimentation as post-analysis revealed problems that rendered the remaining cases unsuitable. Examples include problems with the DICOM files, improper deidentification, missing data, undesired artifacts present in the images, and cases in which there were questions regarding the annotated lesions, typically due to dense tissue and cases in which there was less certainty about the location of the lesion in one of the mammographic views (either CC or MLO). Although the resulting data counts may be relatively small compared to those reported in other ML domains, we note that for mammography applications our “annotated” data counts are within the range of those reported by others using publicly available DBT data sets.^19^

**Table 1:** Number of studies containing CC, MLO, and US image sets.

Data counts: JHM data	Benign	Malignant	Total
Studies collected	456	419	875
Studies annotated	358	198	556
Studies tabulated	54	96	150

Most DBT data from the JHM cases were from Hologic, Inc. mammography systems, though a few (<5) were from other vendors. The average size of DBT slices was 2457×1975  pixels and cubes typically involved 60 to 90 slices. B-mode US data were used, either the radial or anti-radial view.

The second DBT dataset, from the TCIA repository, was used only for training a CNN dual-view x-ray image registration algorithm that is used as part of the system. For this task, a subset of the TCIA data, containing single masses that were biopsy proven, was used to augment the JHM DBT data, thereby increasing the DBT data used to train the registration algorithm as discussed in Sec. 2.5. Table 2 shows the number of TCIA cases that were used. The cases were based on same selection criteria used for the JHM data, except for the requirement of having matched US images and the requirements related to image collection date rate ranges. This resulted in 100 TCIA DBT cases used to augment the JHM DBT data.

**Table 2:** Number of studies used from the TCIA dataset.

Data counts: TCIA data	Benign	Malignant	Total
Studies with annotation	137	87	224
Studies tabulated	60	40	100

Additional details on the usage of the data for training and testing various components of the UDF system are discussed in Table 3. Details are also elaborated upon in the remainder of Sec. 2, following an overview of the system, and in Sec. 3, experimental results are presented.

**Table 3:** Details on data usage for UDF system.

	Phase	JHM	TCIA
Database		JHM (files not publicly available)	TCIA^20^
Type of images		DBT CC and MLO	DBT CC and MLO
		US	
Number of image files used		150 CC and MLO volumes	62 CC and MLO volumes
		150 US images	
Image usage: data conditioner^a^		95 CC and MLO volumes	—
		95 US images	
	Training	85 each^a^ (90% cross val.)	—
	Validation	10 each^a^ (10% cross val.)	—
	Testing	—	—
Image usage: CNN x-ray registration^b^			
	Training	90 CC and MLO volumes	62 CC and MLO volumes
	Validation	54 CC and MLO volumes	—
	Testing	—	—
Image usage: fusion^c^			
	Training	—	—
	Validation	—	—
	Testing	55 CC and MLO volumes	—
		55 US images	

a Data conditioner used only the JHM data. Training and validation involved a 10-fold stratified cross-validation, where the exact data counts used in each fold were obtained by rounding off fractional values resulting from the percentage. No separate test data were used. Test data were reserved for testing the registration and fusion algorithms.

b CNN X-ray image registration used a mixture of JHM and TCIA data. The 152 pairs were chosen such that there were equal counts for malignant and benign lesions (i.e., 76 pair each). The data were subjected to various augmentations, as described in Sec. 2.5, to generate 2280 CC/MLO image pairs for training. Less than the full amount of tabulated JHM and TCIA data were used as a result of balancing the training data with benign versus malignant cases.

c Fusion utilized CC, MLO, and US data for testing. The fusion process does not involve a training step.

### 2.3 Upstream Data Fusion Methodology Overview

**Fig. 2:**
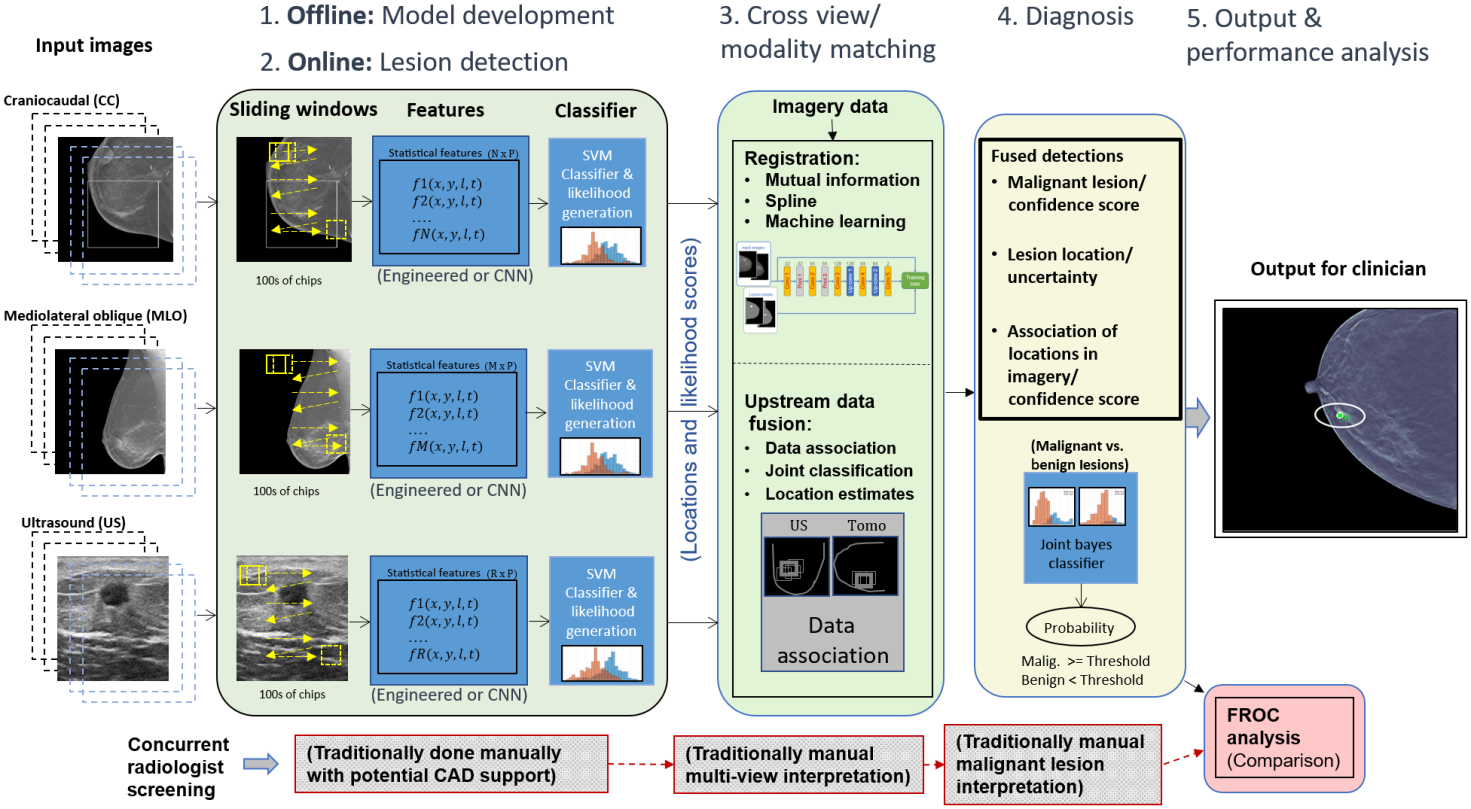
UDF system diagram for breast cancer detection/diagnosis.

The data fusion part of UDF looks back and forth between the images from different modalities and views to automate bringing together (fusing) the different types of relevant information for accurate cancer detection. The objective is to remove false alarms, identify the true lesion (if any), and assign an accurate classification as benign or malignant. The UDF process, as shown in Fig. 2, involves five key steps: (1) statistical model development (done offline), (2) lesion detection (also known as data conditioning), (3) cross-view/cross modality matching, (4) diagnosis, and (5) output performance analysis.

In step 1 (top left in this figure), offline training is done to build statistical feature models for malignant, benign, or other classes of tissue. Steps 1 and 2 involve much of the same algorithmic components. The basic process involves computing image processing features, such as texture measures or transfer-learned convolutional neural network (CNN)-based features from image data. Further, a classifier such as a support vector machine (SVM) or CNN is trained to distinguish the classes. However, in place of using the binary classification output, the process uses the actual decision variable (e.g., distance from decision boundary) to form likelihood values (i.e., a cancer-likeness score) for malignant versus benign lesions.

In step 2, the feature models generated from step 1 are used by data conditioners that process new images from each modality (or view) for the purpose of detecting candidate lesions (i.e., abnormalities). Each candidate detection is assigned a likelihood value based on the statistical models generated from training. It is the set of likelihood values combined with location information (from each modality path) that are provided to the fusion process. Additional details on the statistical feature model development and data conditioners are discussed in Sec. 2.4.

In step 3, registration processes help to map detections from corresponding tissue regions. Registration can also be used as an additional means of filtering out detections from the lesion detection step or for providing uncertainty likelihoods on the mappings of lesions between the CC and MLO views. We have experimented with several registration techniques, ranging from simple linear mappings, which also consider breast-geometry properties, such as distance-to-nipple, to custom CNN-based techniques for registering the multi-view mammography images. A CNN mammographic image registration technique is used in one part of the process for mapping lesion-like detections between the CC and MLO x-ray images for the purpose of helping to filter out false alarms. Additional details on the CNN registration implementation are discussed in Sec. 2.5.

The data fusion part of the process, in step 3, performs data association that associates data from different image modalities (or views) that originate from the same anatomical objects and computes the probability that the data are correctly associated. One of the goals of data fusion methods in general is false alarm rejection to reduce the false alarms from individual sources of candidate detections. In the classic text on data fusion,^21^ it is explained the multiple hypothesis data fusion methods allow successful target detection in higher false alarm environments than other methods.

Multiple hypothesis data fusion methods are applied here for breast cancer lesion targets using DBT (CC and MLO) and US data sources. Data conditioners use ML to produce candidate detection locations and produce likelihood scores for malignant and benign lesion types. Engineered features such as co-occurrence features and features discovered from deep learning can be used in an SVM that is trained to classify lesions as malignant or benign. Our approach goes upstream from the SVM classification and uses the SVM decision variable, the distance to the SVM hyperplane, as a reduced feature. The probability density function of the decision variable conditioned on malignant or benign lesions is estimated as part of ML training as a Gaussian mixture for each data conditioner for CC, MLO, and US. The probability density functions are statistical likelihood functions. The data conditioners for each data source evaluate the likelihood functions for CC, MLO, and US features to compute the likelihood scores for the candidate detections.

UDF uses the data conditioner locations and likelihood scores from upstream SVM decision variables to compute fused detections for lesion location and lesion type as malignant or benign. UDF formulates multiple hypotheses for association of data conditioner detections originating from the same lesions. The CC view is taken as the reference for location data. Registration is performed by linear projection of MLO and US locations into the CC view. Then least squares methods are used to compute estimated lesion location and a 95% error ellipse in the CC view. Future work will integrate CNN-based registration and a nonlinear iterative location estimation algorithm, such as a Gauss-Newton information filter into location estimation as done for other applications in Ref. 17.

Then the methods of Blackman and Reid^21^^,^^22^ are used to form fused detections. The residual fit errors and their uncertainties are used to compute a location hypothesis likelihood score. The likelihood scores for malignant and benign lesions are combined to produce a feature likelihood score. Since lesion location data and feature data are statistically independent, the likelihood scores can be multiplied to produce a combined location and feature likelihood. UDF uses the methods of Blackman and Reid to compute the likelihoods for different hypotheses to find the hypothesis with the maximum likelihood to provide fused detections.

In step 4, the fusion process considers the probabilities of correct association and the probabilities of malignancy, and when both values are high, cancer detection is declared. The radiologist is given not only a signal of a suspicious lesion detection but also an assessment of the probability of the presence of malignancy. Additional details on the underlying theory of the fusion process are discussed in Sec. 2.6.

In step 5, the output of the fusion process is provided as a set of graphical overlays on the x-ray image data, along with quantitative values or probability of malignancy. An optional part of step 5 (shown in red on the lower right side of this figure) is a post-processing analysis step that involves comparing, the performance of a radiologist with the performance of the fusion system for detection and diagnosis via free-response receiver operating characteristic (FROC) curve analysis.

### 2.4 Data Conditioner Implementation and Training Details

In Fig. 2, the lesion detection panel (left-most green panel) for step 2 illustrates how the individual breast imaging modalities and views (e.g., DBT CC, DBT MLO, and US) are first individually processed for supporting the data association and fusion process. For DBT data, individual slices are convolved with a sliding window, for feature computation, as depicted. The US data involved only single images. Sliding window sizes and other hyperparameter settings were selected based on optimization experiments, discussed in Sec. 3.1. For DBT, the sliding window size was set to 250×250  pixels and for US, 150×150  pixels. During training, based on optimization analysis, only sliding window positions that overlap the ground truth lesion locations by at least 65% are considered. (Experiments had shown that, given our data counts, training on lesion locations resulted in better classification performance than training over the entire breast region.) The sliding window positions were also staggered such that multiple feature samplings could be computed for a given lesion area. This also resulted in increased data counts for training. For DBT data, a fixed number of slices, set at 11 slices, centered across the ground truth lesion locations are considered during training. During testing, every $k^{th}$ slice across the entire cube is considered, given the high correlation between adjacent slices, and, again, based on optimization experiments. Further, this minimizes computing and storage requirements. Image processing was employed to mask the breast tissue area of images such that convolution was only applied to tissue areas. For US images, the appearance of the lesions in the images, combined with general lesion location constraints, based on the imaging process, allowed for further bounding of the window location for feature computation.

At each window position, a set of texture features were computed, including co-occurrence features, edge detection-based features, and local intensity values and ratios.^23^ Hence the result of the sliding window operation for each image type was an N×P sized feature matrix (where N was unique for CC, MLO, or US). During training, the ground truth lesion location was also stored in the feature vector. Experiments were also conducted using CNNs for direct pixel-based feature computation. However, given the limited data counts, it was found that engineering features, such as co-occurrence, yielded comparable feature discrimination between benign versus malignant lesions, and other breast tissue areas.

As shown in Fig. 2, feature sets from each modality and view were then subjected to SVMs. As opposed to using the SVMs for binary classification decisions, a process was employed to extract likelihoods of benignness and malignancy based on modeling the distances between the actual support vectors from the SVM hyperplane using a Gaussian mixture model. The likelihoods, versus binary decision outputs, serve as important information components for input to the subsequent data association and fusion steps. A 10-folder stratified cross validation was used for training the data conditioners. Data counts are shown in Table 3.

### 2.5 Automated X-Ray Image Registration Implementation and Training Details

An existing CNN-based mammographic image registration technique, described in Ref. 24, was used for aiding in the process of matching detections between the DBT CC and MLO views, which in turn supported false alarm reduction. The registration involves a deformation field-based CNN technique for registering features between CC and MLO x-ray mammography images. For our experiments, the network was trained using 152 pairs of DBT volumes, each containing a single lesion. The 152 DBT volumes involved a mixture of DBT data from the JHU and TCIA data sets. Further, this set excluded DBT images that were designated as “test” images from the original 150 JHM cases used in the overall study. There was an equal proportion of malignant and benign lesions (i.e., 76 DBT pairs for each type). For each DBT volume, five image slices were selected, which intersected the given lesion. Further, two additional augmentations were created for each slice by applying up ±12  deg rotations in addition to the original orientation. Hence, this resulted in 2280 image pairs for training (i.e., 152×5×3). A separate set of 54 images were used for validation during the training. The training and validation data counts are also shown in Table 3.

**Fig. 3:**
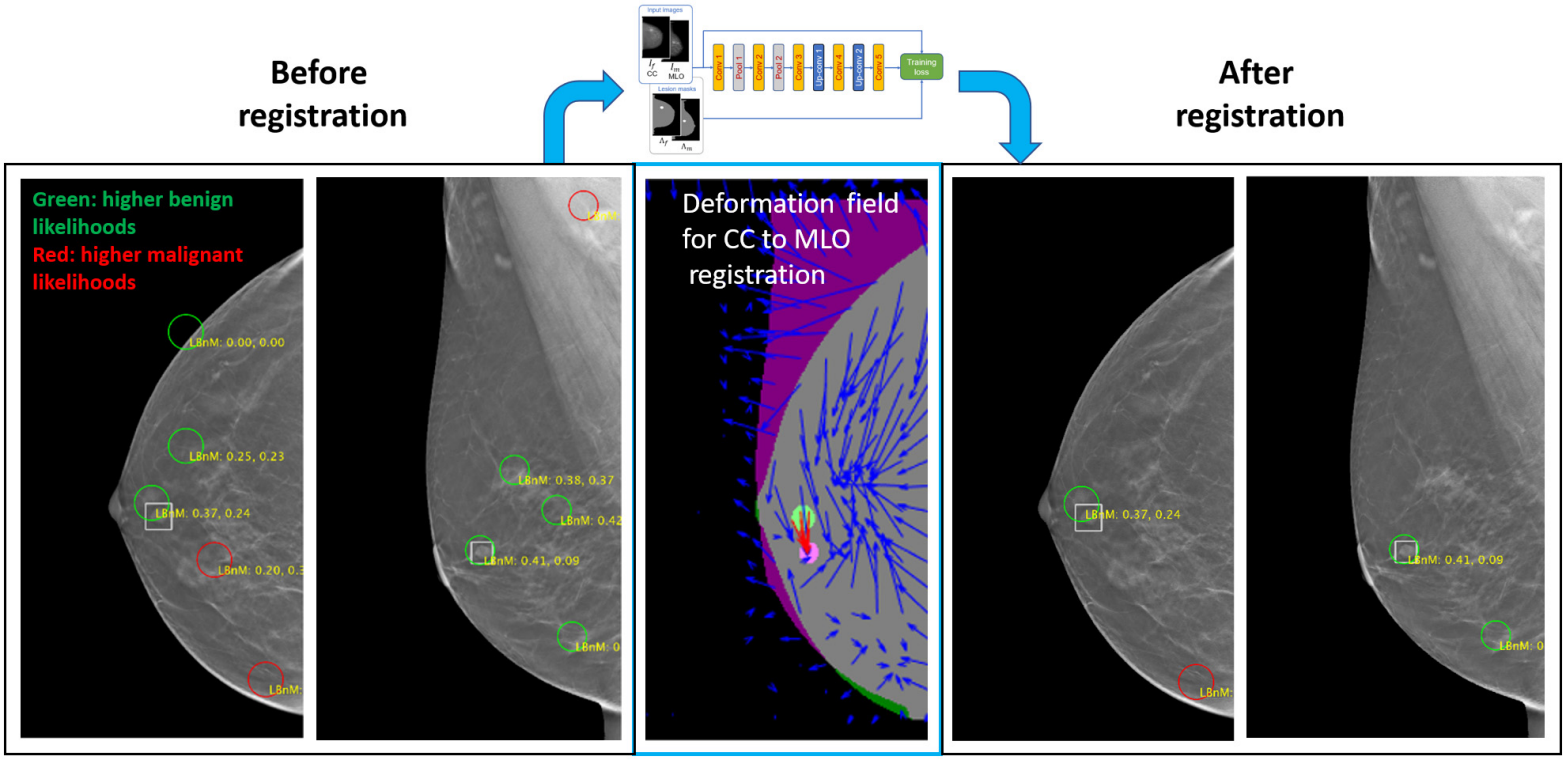
Use of CNN-based CC to MLO registration for reducing candidate detections.

Figure 3 illustrates the usage of the CNN-based registration. The left-most panels show a CC and MLO mammogram pair. The green and red circles denote candidate detections (i.e., potential lesions) on the images. The true lesion location is depicted by the white square, near the nipple. There are five candidate detections in both the CC and MLO views. The middle panel shows a deformation field that was generated by the CNN network. The deformation field provides a mapping between lesion-like tissue between the CC and MLO views. (The network is trained to match areas that are likely lesions versus the tissue in general.) The solid green and solid purple circles on the middle panel represent the lesion in the CC and MLO views, respectively. Hence, the red arrows show that the network accurately mapped the CC lesion pixels to the MLO lesion pixels.

**Fig. 4:**
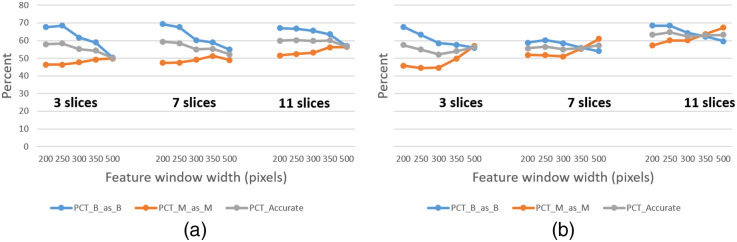
Lesion detector hyperparameter optimization for (a) DBT CC and (b) DBT MLO data.

By applying registration to each CC detection and comparing the resulting mapping location in the MLO view, a means for reducing false alarms was employed. Specifically, if the mapping for a CC detection did not correspond to the general area of one of the MLO detections, then both detections were rejected. For our dataset, in most cases, this resulted in some degree of false alarm reduction, while preserving the lesions. For example, Fig. 4(b), there are only two candidate detections in each view and the lesion is preserved. We note that using this methodology, in a few cases, the lesion may also incidentally be removed by the filtering. In future research, the combination of improved training, based on additional datasets, and the use of confidence measures from the registration, are among the areas that are envisioned for helping mitigate these occurrences.

### 2.6 Details on Upstream Data Fusion Theory

As mentioned earlier, data association is a key part of the fusion process. Moreover, data association is conducted as part of a broader process called multiple hypothesis tracking (MHT).^21^^,^^22^ Data association takes object candidate detections from each modality (or view) and forms associations of the candidate detections to provide object candidate associations. Depending on the overall number of detections and the number of sensors or views, many different combinations of associations may be formed, though some are filtered out due to certain constraints, such as not meeting certain location criteria. For example, a candidate detection that is close to the nipple in the CC view may be prevented from being associated with a candidate detection near the pectoral muscle in the MLO view.

MHT is an iterative process that considers an initial set of object candidate associations as well as various additional child and parent sets of associations that are considered in subsequent iterations. The key algorithmic components of MHT are reported by Reid^22^ and are shown in Reid’s Eqs. (1) and (2). The equations involve a form of a Bayes posterior probability computation. To aid in explaining the terms, we present an analogy using our subject domain, breast imaging. Suppose a set of object candidate detections was detected from a DBT-CC view (e.g., three), another set was detected from a DBT-MLO view (e.g., two), and a set was detected in a US image (e.g., two). Suppose that there is only one lesion (or abnormality). In simple terms, the goal of MHT is to associate object candidate detections into an object candidate association with a location and probability of malignancy of the lesion.

The MHT process starts with object candidate detections from the first image file, the CC view in our analogy. Each of the initial CC detections constitutes a measurement. Further, it is assumed that each detection is a unique object (abnormality) or it is a false alarm. When the next modality or view (e.g., MLO) is processed, detections resulting from it constitute new measurements (potentially on the same object, or abnormality, that was detected in the first image). This new set of detections also constitutes the next iteration of the MHT process. Barring various other conditions that could occur, it is at this point that the first set of associations can be formed. For each object candidate association at this iteration (i.e., combination of CC and MLO detections), the posterior probability is computed by Eqs. (1) and (2). We note that at this point, the actual iterative logic in MHT can vary due to different additional hypothetical means of forming associations such as the formation of multiple object associations (e.g., the association of sets of measurements across iterations). For simplicity’s sake, here we assume that the next iteration involves simply the set of new measurements from the US data. It is at this point that parent object candidate associations from previous data (CC and MLO), denoted by $\Omega_{g}$ in Eqs. (1) and (2), can be formed. Furthermore, the association of $\Omega_{g}$ with the US-based object candidate detections are child object candidate associations, denoted by $\Psi_{h}$ in Eqs. (1) and (2). For each object candidate association at this iteration (i.e., combination of CC, MLO, and US detections), the posterior probability is computed by Reid’s Eqs. (1) and (2).

The sequential process involves step-by-step evidence accrual in that through each iteration k (where k=1 for CC, k=2 for MLO, and k=3 for US) probabilities for certain associations are formed. Associations can be expanded or pruned and eventually the best hypotheses are retained:

$$P(\Omega_{i}^{k}|Z^{k})=P(\Psi_{h},\Omega_{g}^{k-1}|Z(k),Z^{k-1}),$$ (1)

$$P(\Psi_{h},\Omega_{g}^{k-1}|Z(k),Z^{k-1})=\frac{P(Z(k)|\Psi_{h},\Omega_{g}^{k-1},Z^{k-1})P(\Psi_{h}|\Omega_{g}^{k-1},Z^{k-1})P(\Omega_{g}^{k-1}|Z^{k-1})}{P(Z(k)|Z^{k-1})},$$ (2)

where $\Omega_{i}^{k}$ is the multiple object association $\text{hypothesis}=\{\Psi_{h},\Omega_{g}^{k-1}\}$; $\Psi_{h}$ is the child data association hypothesis; $\Omega_{g}^{k-1}$ is the parent hypotheses through k−1 scans; $Z(k)=\{z_{1}(k){,z}_{2}(k),\ldots {,z}_{m_{k}}(k)\}$ is the set of measurements on the current scan at time $t_{k}$; and $Z^{k}$ and $Z^{k-1}$ are all measurements in scans through scan k and k−1, respectively.

In Reid’s equations, the reader will note that the right side of Eq. (1) is expanded in Eq. (2). The left side of Eq. (2) is the probability of the data association hypotheses through the k’th sequential scan where a scan is the set of measurements from one modality or view. Here scan is not a medical imaging term but a term from data fusion methods. Scan refers to data from data sources that satisfy the scan constraint for each association hypothesis of parents and children that for each parent there is at most one child and for each child there is at most one parent. The scan constraint greatly limits the number of hypotheses that need to be considered. Also it allows the use of optimal assignment algorithms such as the Hungarian algorithms to find the best association hypotheses.^21^

The data association hypothesis is the combination of the child hypothesis h for new measurements with the parent hypothesis g for all previous measurements $Z^{k-1}$ in scans through k−1. On the right side of Eq. (2), in the numerator, the first term represents the conditional likelihood function for the new measurements for the data association hypothesis. This term is obtained by multiplying the location likelihood and the feature likelihood terms as discussed in Sec. 2.3. The second term is the probability of the child hypothesis given the parent hypothesis and data through scan k−1. This is determined as a binomial distribution from the probability of detection (PD) of the ML detection algorithms. The third, final term is the probability of the parent multiple object association hypothesis from the last iteration. The denominator term provides normalization. The reader is referred to Refs. 21 and 22 for additional details on the MHT process.

## 3 Results

Results from applying the UDF system for lesion detection and classification using DBT CC, DBT MLO, and US images are discussed in this section. Details on the performance of the data conditioners and the CNN x-ray image registration are also provided.

### 3.1 Data Conditioner Performance (for Individual Views and Modalities)

As discussed in Sec. 2.4, the data conditioners (i.e., lesion detectors) for DBT and US were subjected to optimization experiments for determining hyperparameter settings, such as feature window sizes. Figure 4 shows the performance (average cross validation results) that was yielded for different feature window sizes and different numbers of slices (through ground truth lesions) processed by the DBT CC and MLO data conditioners. Similar optimization analysis was performed for the US data conditioner. Feature window size widths of 250 and 150 pixels for DBT and US, respectively, were selected in large part based on the optimization analysis. For DBT, the data conditioners were configured such that feature windows were applied to 11 slices, centered about the ground truth lesion locations during training. However, during test time, feature windows were applied to slices spaced k=5 slices apart throughout the entire breast tissue. This value was chosen based on similar performance analysis and also based on computing resource limitations. A next step during the test time processing is to apply a hierarchical clustering to combine neighboring detections which likely constitute the same tissue (e.g., lesion). Hence, in this manner, tissue from across the entire breast is processed.

Table 4 shows the classification accuracies achieved by the data conditioners when using a 10-fold, stratified cross validation, based on 95 lesions (50 malignant and 45 benign) [see Table 3]. The DBT-based classification accuracy values are comparable to sensitivity values reported by other basic lesion detection algorithms on DBT.^19^ While more advanced lesion detection capabilities may provide higher detection rates, the fusion of multiple views and modalities also yields high detection rates, with lower false alarms, and improved lesion location estimation, as demonstrated in Sec. 3.3. Hence, our system can work with a variety of available lesion detection systems and yield improvements when either multi-view or multiple modality images are available.

**Table 4:** Data conditioner cross validation performance—individual modalities/views.

	CC accuracy (60%)		MLO accuracy (61%)		US accuracy (69%)	
	Classified as		Classified as		Classified as	
	B	M	B	M	B	M
B	69.1	30.9	62.5	37.5	70.4	29.6
M	48.9	51.1	39.6	60.4	31.3	68.7

Note: B indicates benign and M indicates malignant.

### 3.2 CNN Registration Performance

**Fig. 5:**
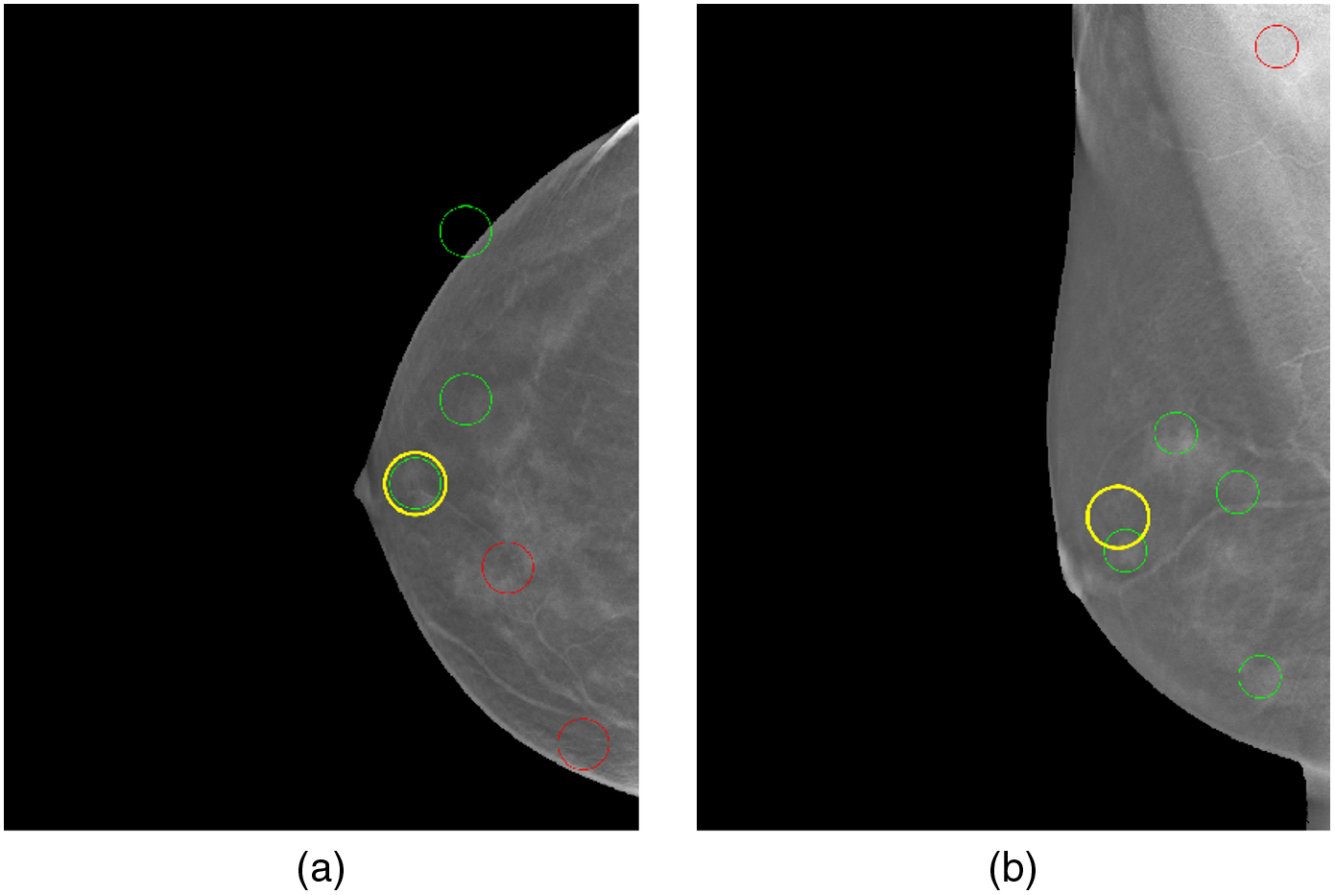
Illustration of (a) CC and (b) MLO registration. In (a), a yellow circle is shown over the ground truth annotation for lesion in the CC view. In (b), the yellow circle, positioned at the centroid of the displaced CC pixels is shown to intersect the MLO lesion ground truth.

As discussed in Secs. 2.3 and 2.5, the CNN registration was used to help filter out excessive false alarm detections from the CC and MLO image data conditioners prior to the fusion process. Referring to Fig. 5, the criteria for successful registration was as follows. First, the centroid of all displaced pixels from the CC lesion ground truth region was computed. Next, a circular region, adjusted slightly larger than the CC lesion ground truth, was centered at the centroid location of the mapped CC pixels, which resulted from the registration. If the displaced circular region intersected the corresponding ground truth for the MLO lesion, then the registration was considered successful. This criterion was used to filter detections from the CC and MLO images for passing to the fusion process. Using this convention on the test data, the CNN registration demonstrated a 94% success rate at matching lesions between the CC and MLO views (for cases where the lesion was detected in all modalities). Further, it achieved a 44% false alarm reduction rate, which translated to reducing the number of false alarms, on average, from 7.09 to 3.95 per image (for CC and MLO). In one test scenario, this was a decrease from 156 to 87 false alarms across the test images. In the current system design, the CNN registration results in a small percentage of false negatives for lesions. However, in future designs, the registration mapping information, along with all detections, would be passed into the fusion process, where fusion could then use the additional information to further aid in reducing false alarms.

### 3.3 Fusion Results

The UDF test set consisted of 55 cases (Table 3). The focus of the fusion results is to present cases and overall statistics that show how fusion using UDF can reduce ML candidate detections to useful fused detections. ML detections from CC, MLO, and US may provide an ambiguous and complicated set of data with excessive false alarms (ML yields up to 5 detections per view, so there may be up to 15 detections per case, many of which are false positives or false alarms). It is a goal of UDF to combine the ML data to provide a product with an improved false alarm rate (FAR). Eight cases are presented here with overall statistics that illustrate the improvement that UDF can produce and also show limitations in UDF performance. Further research is described that can help to remove the limitations.

**Fig. 6:**
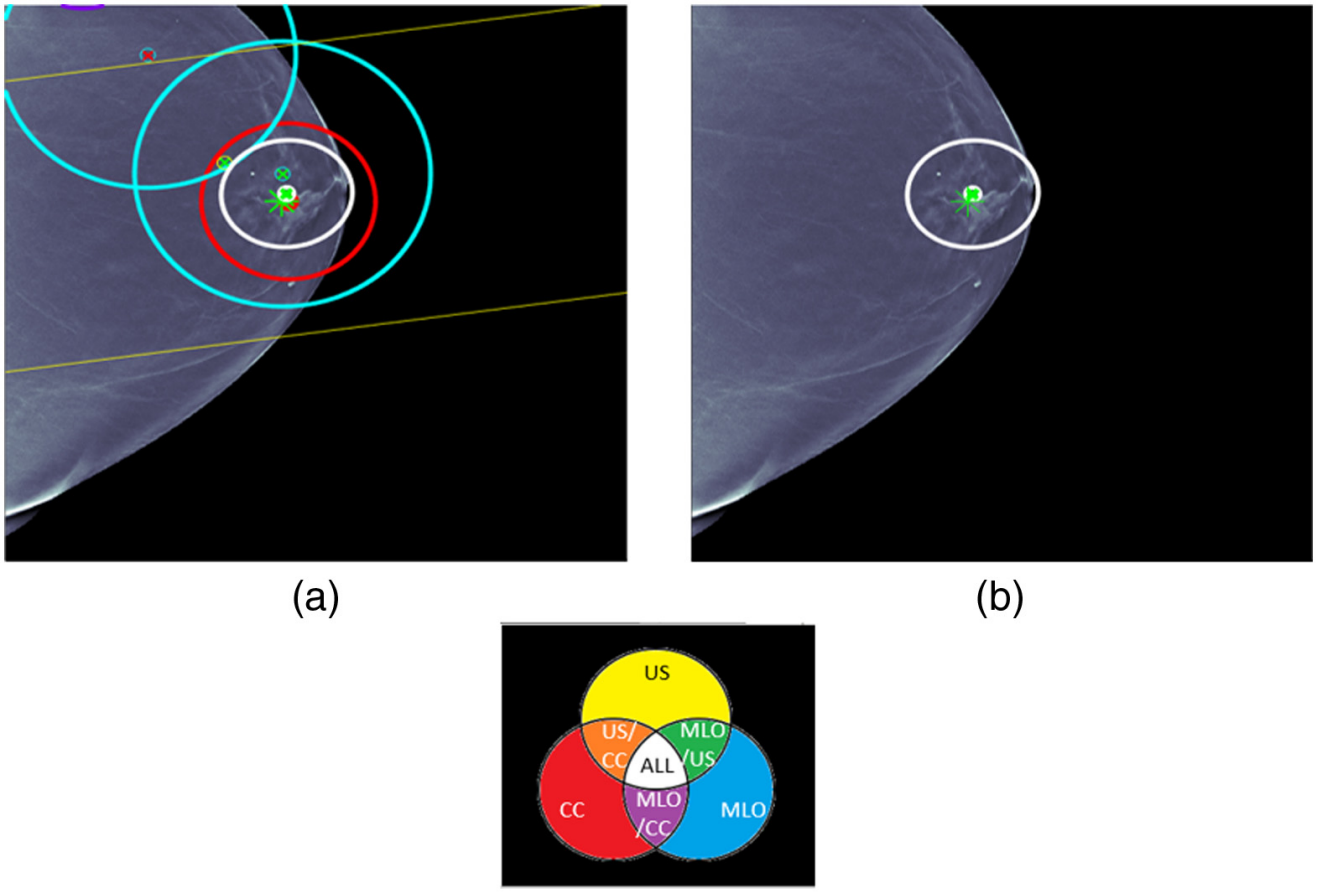
UDF case 1: (a) data conditioner detections projected onto the CC view and (b) the fused output. The color wheel at the bottom shows the color scheme for the ellipses and graphics in (a) and (b). The data conditioner detections and fused detection are indicated by ellipses according to the color combinations. Centers of detections have red x for malignant and green x for benign. Ground truth lesion indicated by asterisk that is red for malignant and green for benign. The same labeling is used for all cases.

Case 1 is provided in Fig. 6. ML detections and the fused detection are shown with location error ellipses according to the color combination scheme shown in this figure. For example, the red ellipse is an ML detection for CC and the two blue ellipses are ML detection for MLO. The pair of yellow lines corresponds to a long yellow ellipse for an ML detection for US that is due to the fact that only clock face and nipple distance are used for the ML US detection location. The white ellipse is the fused detection that combines CC, MLO, and US detections. The centers of the ellipses are red for malignant detections and green for benign detections. The ground truth for the lesion is indicated by an asterisk that is red for malignant and green for benign. The same labeling is used for all the cases. For each figure, the image on the right displays the fused detection, and the image on the left shows the ML detections that were used in forming the fused detection. Some later cases will have more than one fused detection. UDF is a multiple hypothesis MHT method, and the fused detections that are shown are for the statistically best hypothesis that uses the CC, MLO, and US image types.

Case 1 is a relatively straightforward case where there are CC, MLO, and US ML detections that localize and classify the lesion correctly. There is only one ML false alarm for an MLO ML detection. UDF easily provides a single fused detection that localizes and classifies the lesion correctly.

**Fig. 7:**
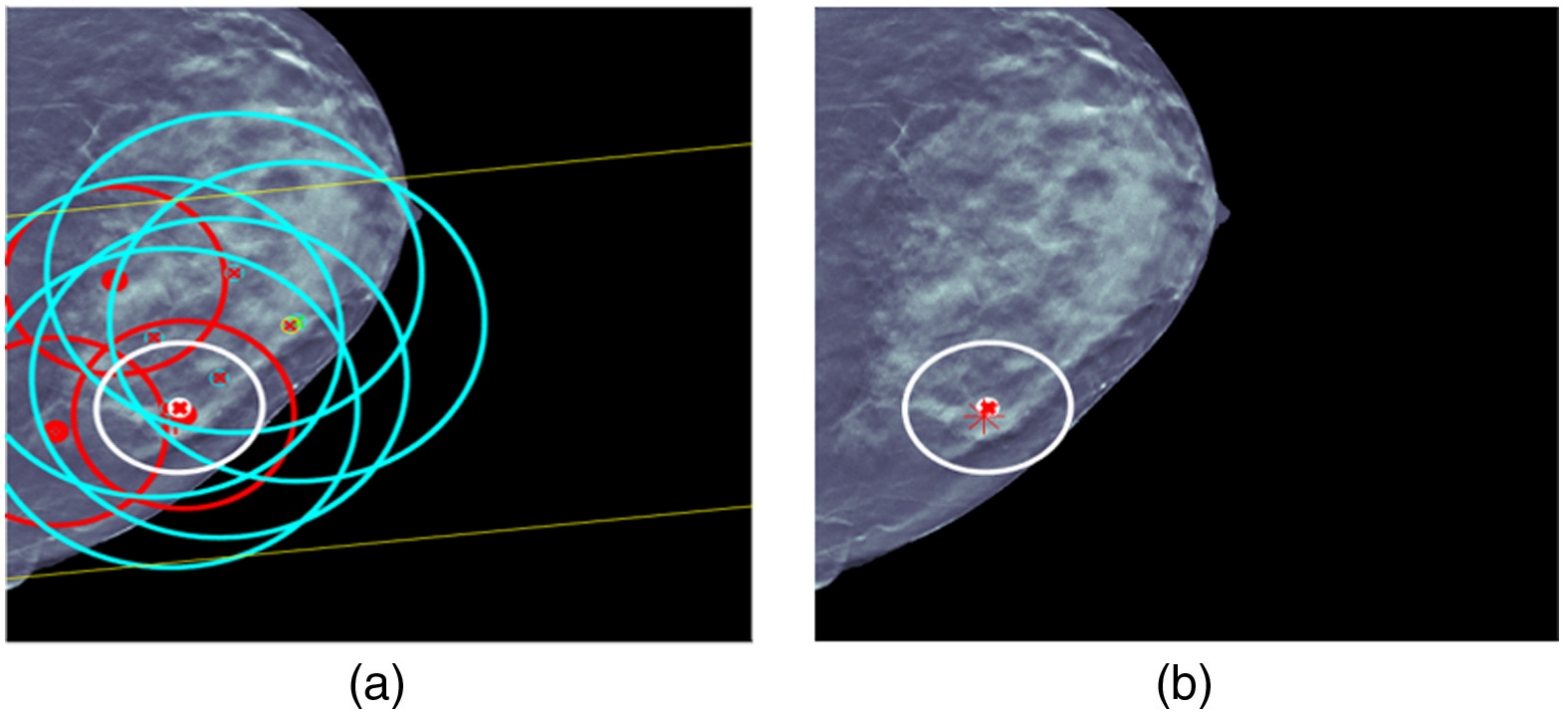
UDF case 2: (a) data conditioner detections projected onto the CC view and (b) the fused output.

For case 2 in Fig. 7, the situation is more complicated. There are a number of ML detections of questionable usefulness, and UDF reduces the ML detections to a single fused detection that localizes and classifies the lesion correctly. This shows the kind of behavior of multiple hypothesis methods that has been observed in other types of applications, such as military tracking of targets. In military applications, it has been observed that multiple hypothesis MHT methods can operate successfully in false alarm environments that are an order of magnitude larger than tolerated without multiple hypothesis methods.^21^

**Fig. 8:**
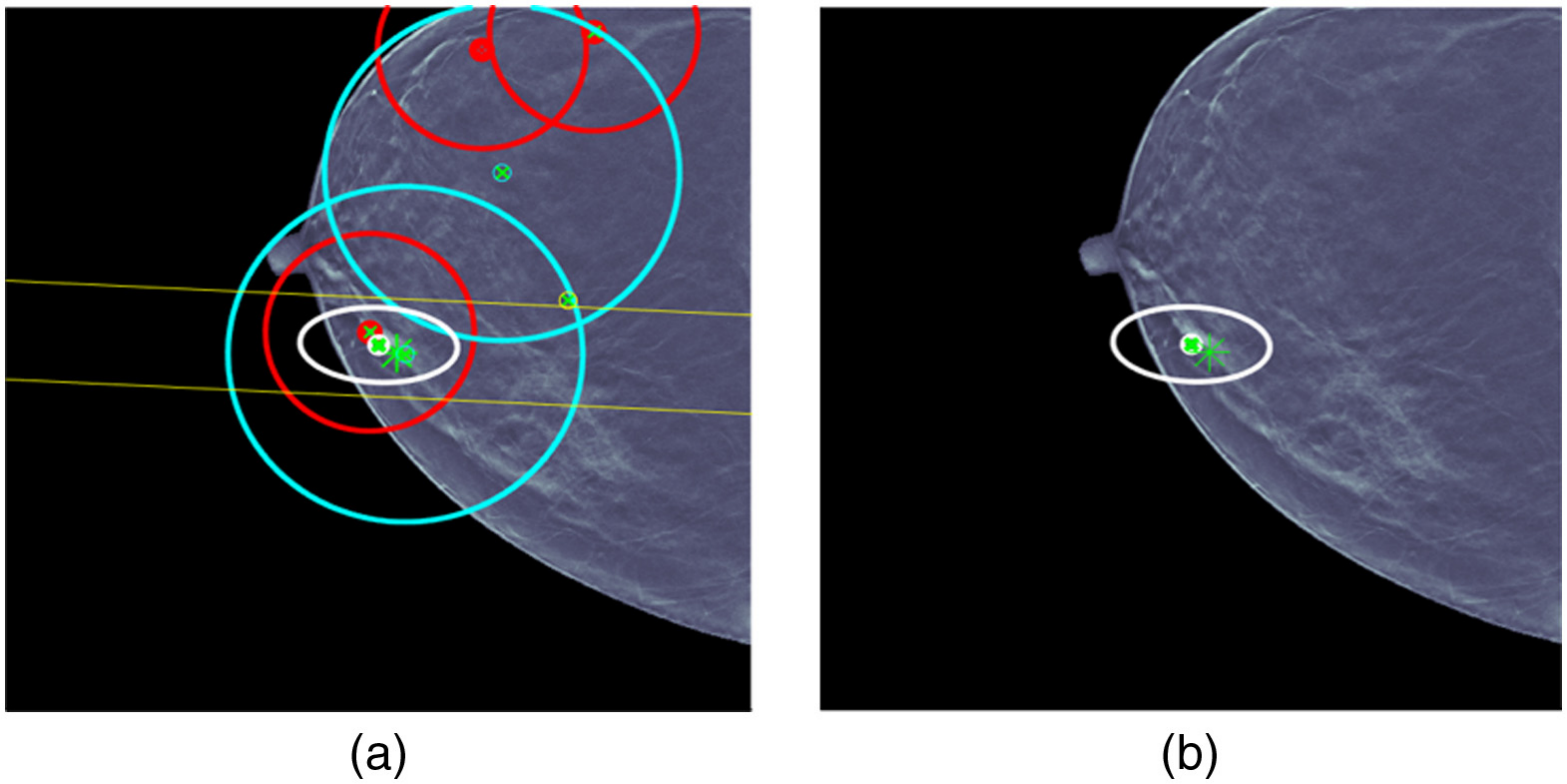
UDF case 3: (a) data conditioner detections projected onto the CC view and (b) the fused output.

**Fig. 9:**
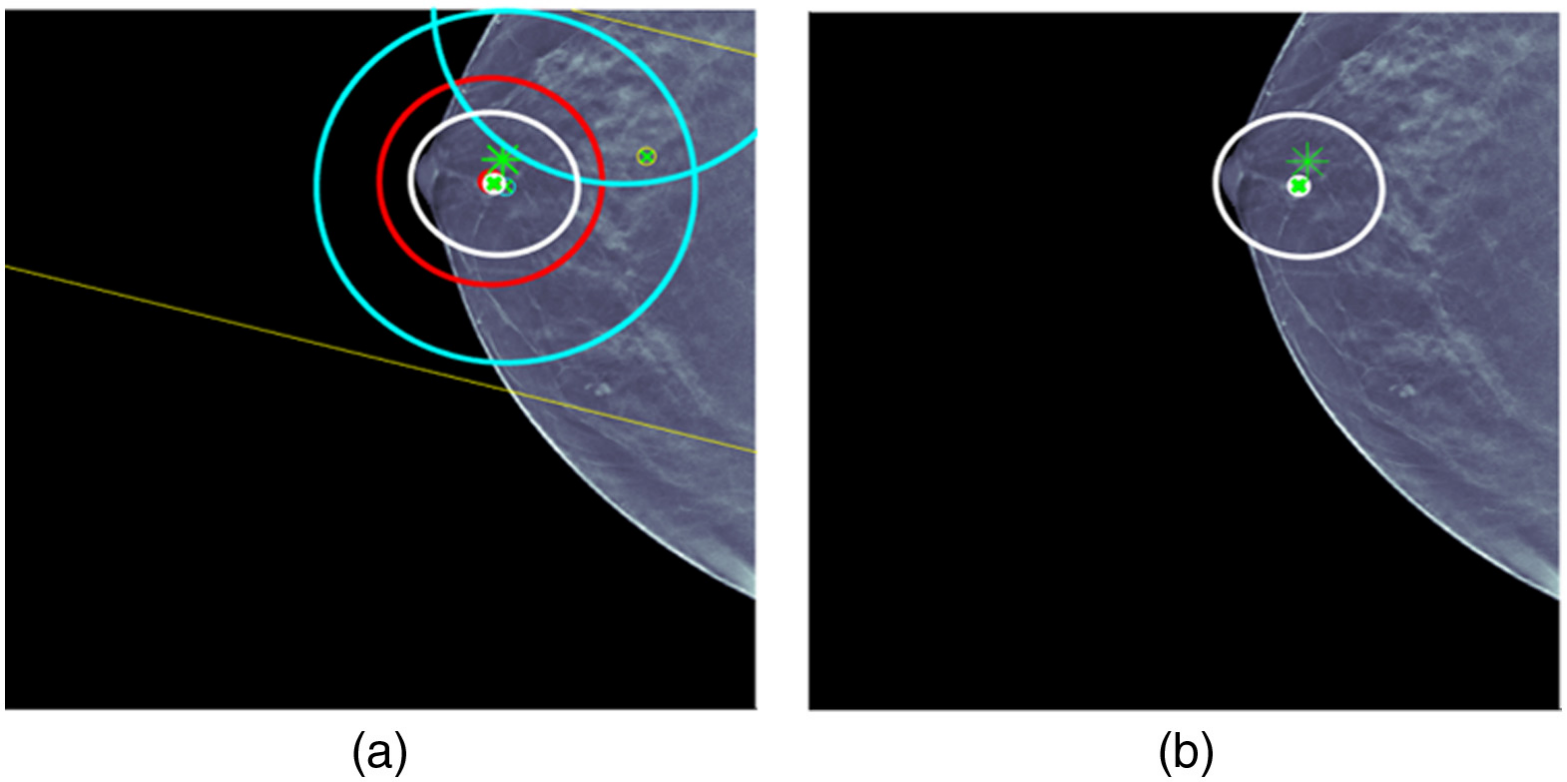
UDF case 4: (a) data conditioner detections projected onto the CC view and (b) the fused output.

For cases 3 and 4 in Figs. 8 and 9, respectively, the situation is less complicated and a single fused detection is produced that localizes and classifies the lesion correctly. For case 3, ML detection false alarms would seem to provide an opportunity for a fused detection false alarm made up of CC and MLO ML detection false alarms, but UDF correctly interprets the evidence.

**Fig. 10:**
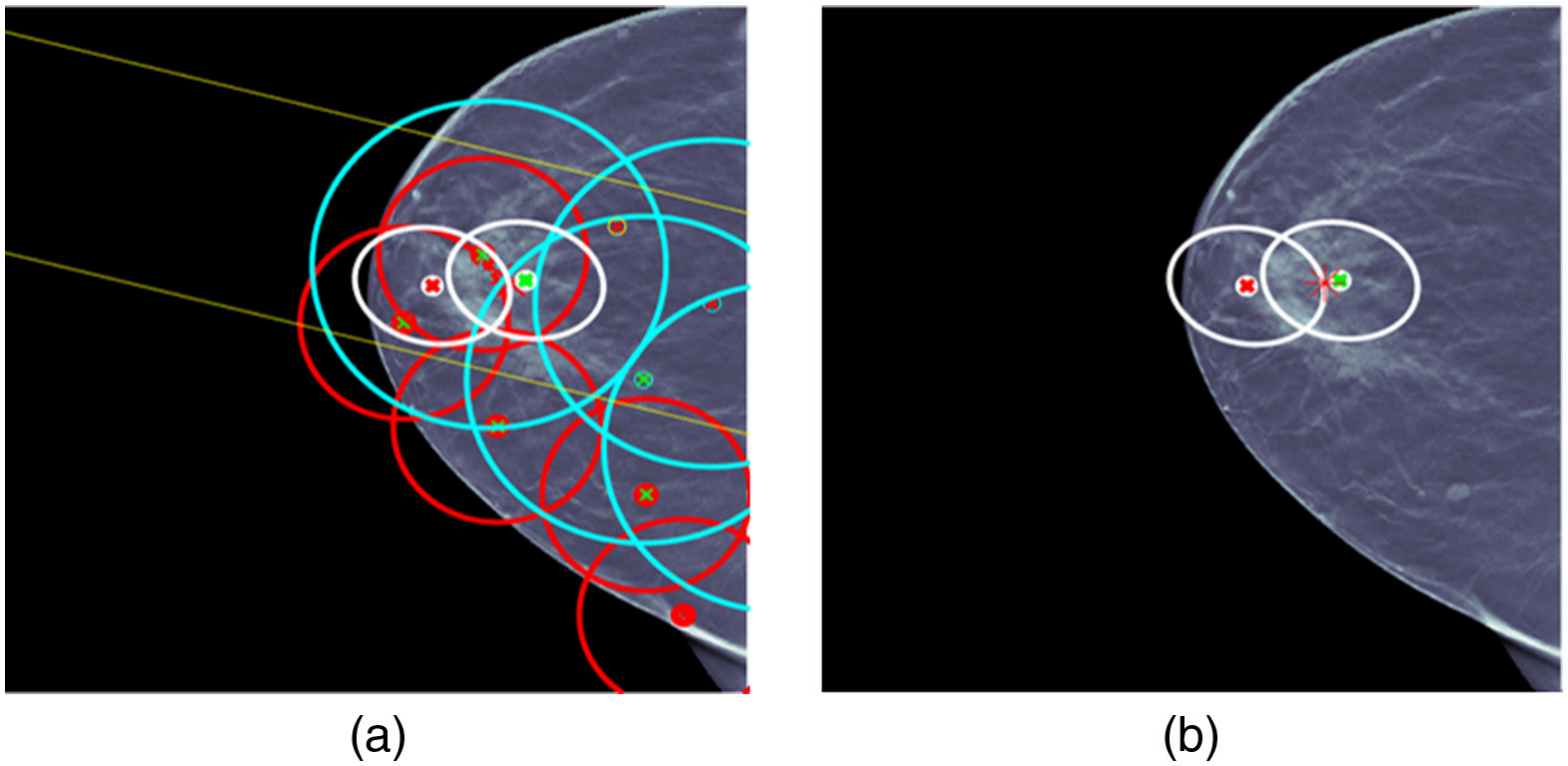
UDF case 5: (a) data conditioner detections projected onto the CC view and (b) the fused output.

For case 5 in Fig. 10, the situation is again complicated. There are a number of ML detections of questionable usefulness. UDF produces an incorrect fused detection with an ellipse that contains the lesion but is classified incorrectly. The CC ML detection in the fused detection was classified incorrectly so this appears to be due to a limitation in ML classification. UDF also reduces the ML detections to an incorrect fused detection that localizes the lesion just outside the edge of the ellipse and classifies it correctly. This could be due to a limitation in registration. Full integration of the CNN-based image registration approach described earlier and extension to US data could help remove this limitation. The current registration is performed with a linear transformation of MLO and US detections into CC as shown in the left-hand-side images for all the cases. The MLO blue ellipses are always bigger than the CC red ellipses due to the observed uncertainty in the transformation. The CNN-based registration with a nonlinear deformation field would better account for different breast positions and effectively reduce the size of the blue ellipses. This would be accomplished using a nonlinear iterative location estimation algorithm, such as a Gauss–Newton information filter that we have used in other applications, such as military tracking.^17^ With the improved uncertainty, there would be less opportunity for false alarms.

**Fig. 11:**
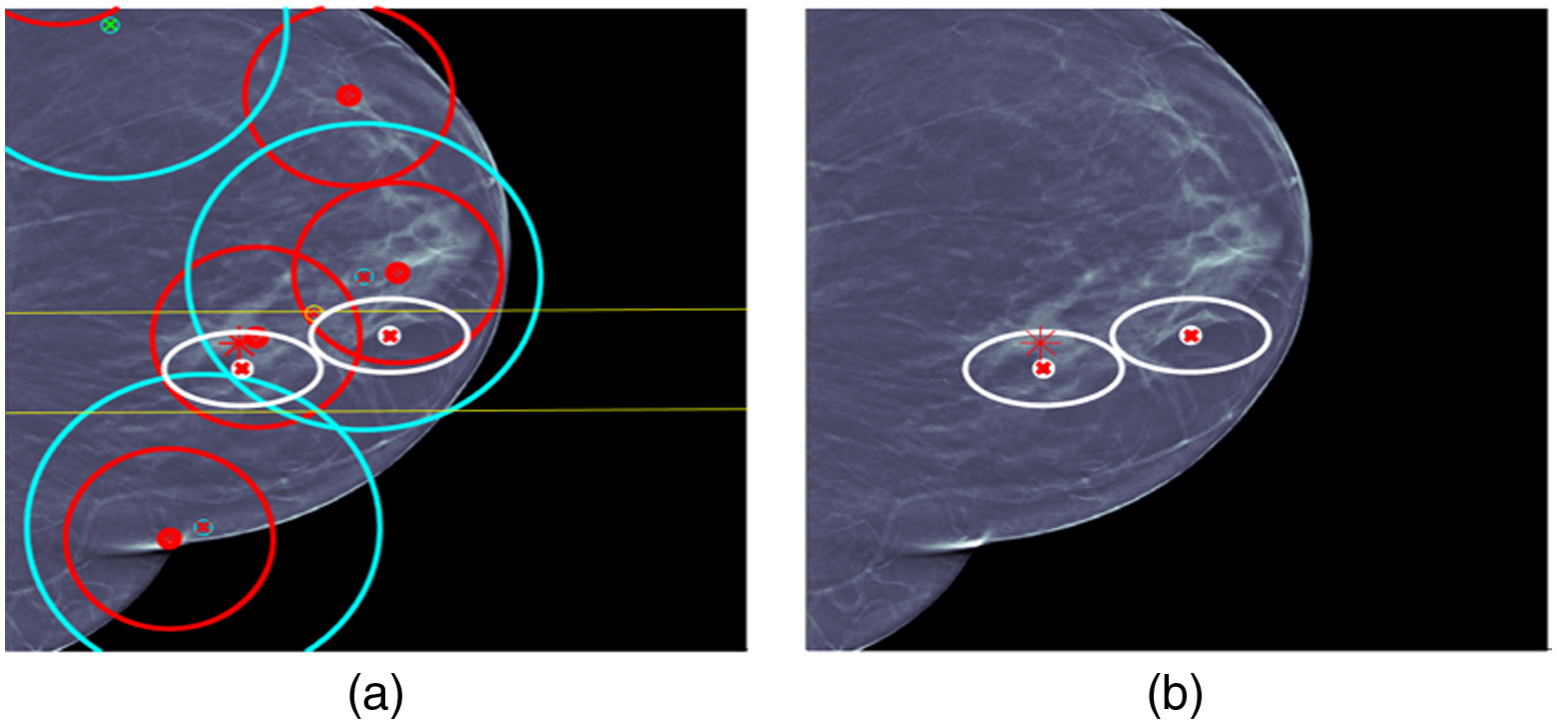
UDF case 6: (a) data conditioner detections projected onto the CC view and (b) the fused output.

For case 6 in Fig. 11, the situation is also complicated. Again, there are a number of ML detections of questionable usefulness. UDF reduces the ML detections to a fused detection that localizes the lesion and classifies it correctly. UDF also shows a limitation by producing a fused detection false alarm with an ellipse that is near the correct fused detection and classified malignant. The same type of improvements as described for case 5 could help to remove the limitation. It is worth noting that there would seem to be opportunities to form false fused detections using CC and MLO ML detections, but UDF resists forming the fused detections.

**Fig. 12:**
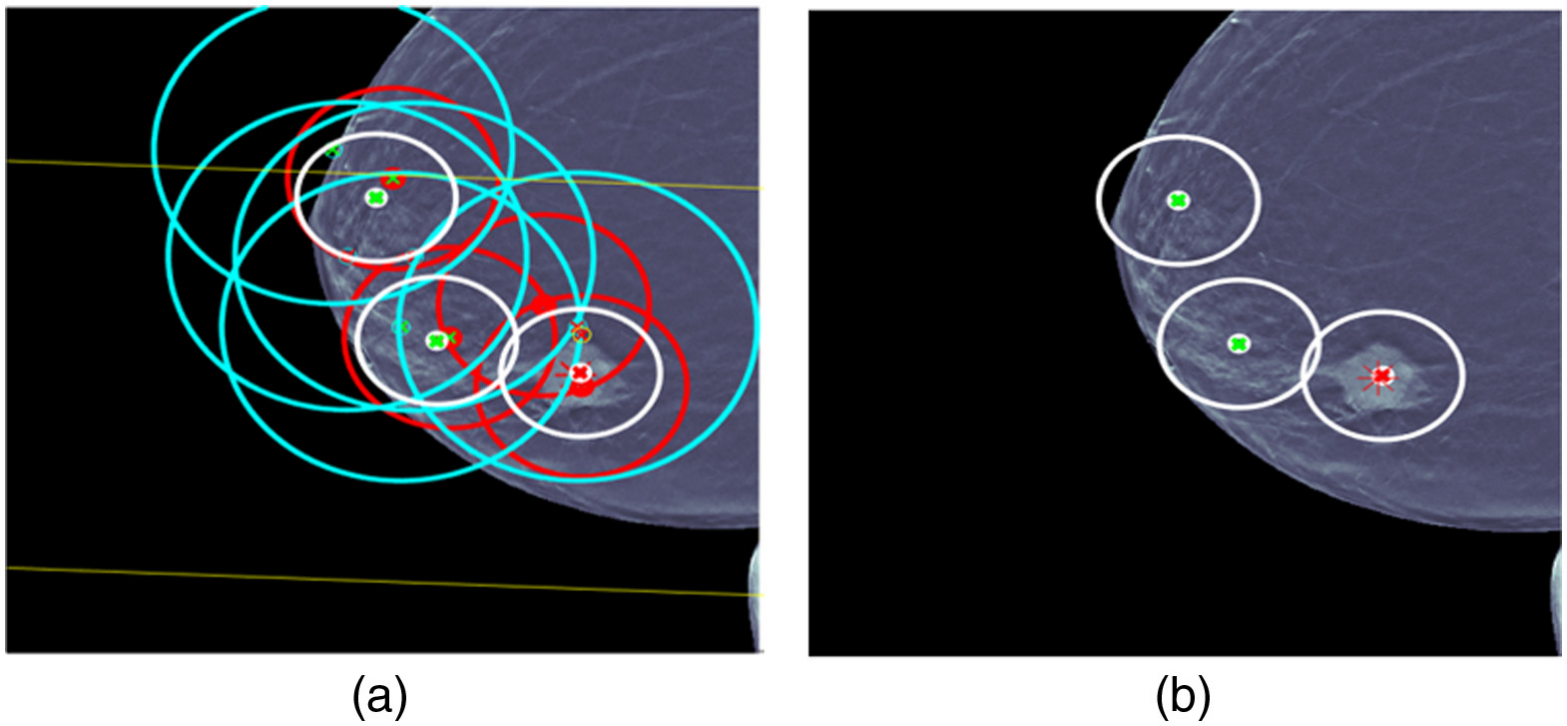
UDF case 7: (a) data conditioner detections projected onto the CC view and (b) the fused output.

For case 7 in Fig. 12, there is another complicated situation. Again, there are a number of ML detections of questionable usefulness. UDF reduces the ML detections to a fused detection that localizes the lesion and classifies it correctly. UDF also shows a limitation by producing two fused detection false alarms that are classified benign. The same type of improvements as described for case 5 could help to remove the limitation.

**Fig. 13:**
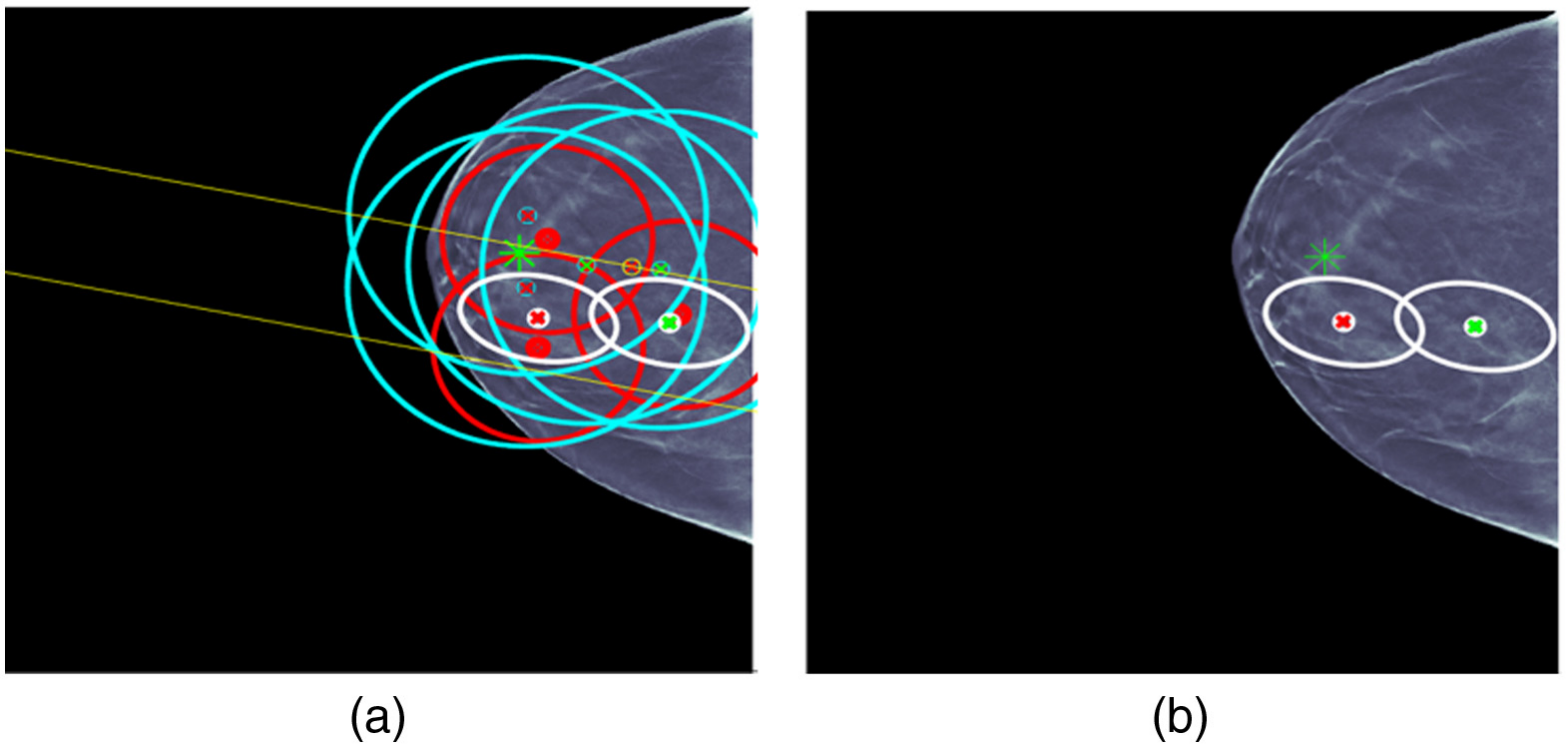
UDF case 8: (a) data conditioner detections projected onto the CC view and (b) the fused output.

For case 8 in Fig. 13, there is another complicated situation. Again, there are a number of ML detections of questionable usefulness. This time UDF does not reduce the ML detections to a fused detection that localizes the lesion and classifies it correctly. UDF shows a limitation by producing two fused detections that do not contain the lesion in the error ellipse where one is classified malignant and the other benign. The lack of containment could be due to registration limitations. Also several CC and MLO ML detections that are classified incorrectly as malignant contribute to the fused detection that is malignant, and this is a limitation of ML classification.

One way to evaluate detection approaches is to use the FROC curve. In the data fusion community, multiple hypothesis methods using Reid’s equations described in Sec. 2.5 take as input from each source of data the parameters for probability of detection (PD) and FAR per unit volume of sensor space.^21^^,^^22^ But PD and FAR are the same as sensitivity and average false positives per unit volume from a point on an FROC curve. There are extensive sets of fusion metrics implemented in a number of tools to evaluate data fusion approaches.^21^ FROC analysis is often used to evaluate the detection systems that feed into data fusion but not for data fusion itself. However, it seems that evaluation of UDF for a radiological application should include FROC analysis.

The FROC concept is extended here to evaluate UDF. For radiology, the FROC curve can be based on proximity of a detection to a lesion. For example, this was done in the development of the baseline for the DBTex effort for breast imaging.^19^^,^^25^ A true positive was defined as a detection that satisfies a proximity condition to a lesion ground truth. Then an FROC curve was defined as the plot of true positive fraction (sensitivity) versus average false positives per DBT volume. Attempting to extend this approach to UDF, it seems natural to base it on a proximity condition that the UDF detection contains the lesion within the 95% error ellipse computed by UDF. But this would not evaluate a benefit of UDF to perform joint classification of a lesion by combining classification information in ML detections over CC, MLO, and US views. Consequently, a better way to define a true positive is a fused detection that contains the lesion in the 95% error ellipse and classifies the lesion correctly as malignant or benign. The false positives then can be defined as fused detections that do not contain the lesion or contain the lesion and misclassify the lesion.

The benefits of UDF are the ability to improve ML detection information by false alarm rejection, joint classification using ML decision variables, and providing 95% error ellipses for fused detections. But to evaluate these benefits, the ML detections must contain true detection amid the false alarms, or no amount of false alarm rejection will produce a correct detection with improved classification and an error ellipse. As a result, the cases that are used to perform the UDF FROC analysis are limited to those that have ML true detections in the midst of false alarms. Now some general comments about UDF performance can be made in the following.

• Of the 55 tabulated studies that were not used for training and validation for ML models, there are 22 cases with true ML detections for CC, MLO, and US views with an average of 8.0 false alarms over all three views.
• For these 22 cases, UDF sensitivity is 90.9% (20/22) and the average number of false positives per case is 1.1. This is for a true positive that contains the lesion and classifies it correctly as malignant or benign.
• This shows performance for false alarm rejection, joint classification, and containment by 95% error ellipses.

The two cases out of the 22 above that did not produce correct fused detections are displayed in Fig. 10 for case 5 and Fig. 13 for case 8. For case 5, it is explained above that UDF produces incorrect fused detections due to limitations in ML classification and limitations in registration. These limitations can be remedied using more sophisticated ML algorithms and using CNN-based registration. For case 8, it is explained above that UDF also produces incorrect fused detections due to limitations in ML classification and limitations in registration. It should be noted that it is not necessary to have true ML detections in all three views to have a correct fused detection, but that will provide the most consistent benefit.

**Fig. 14:**
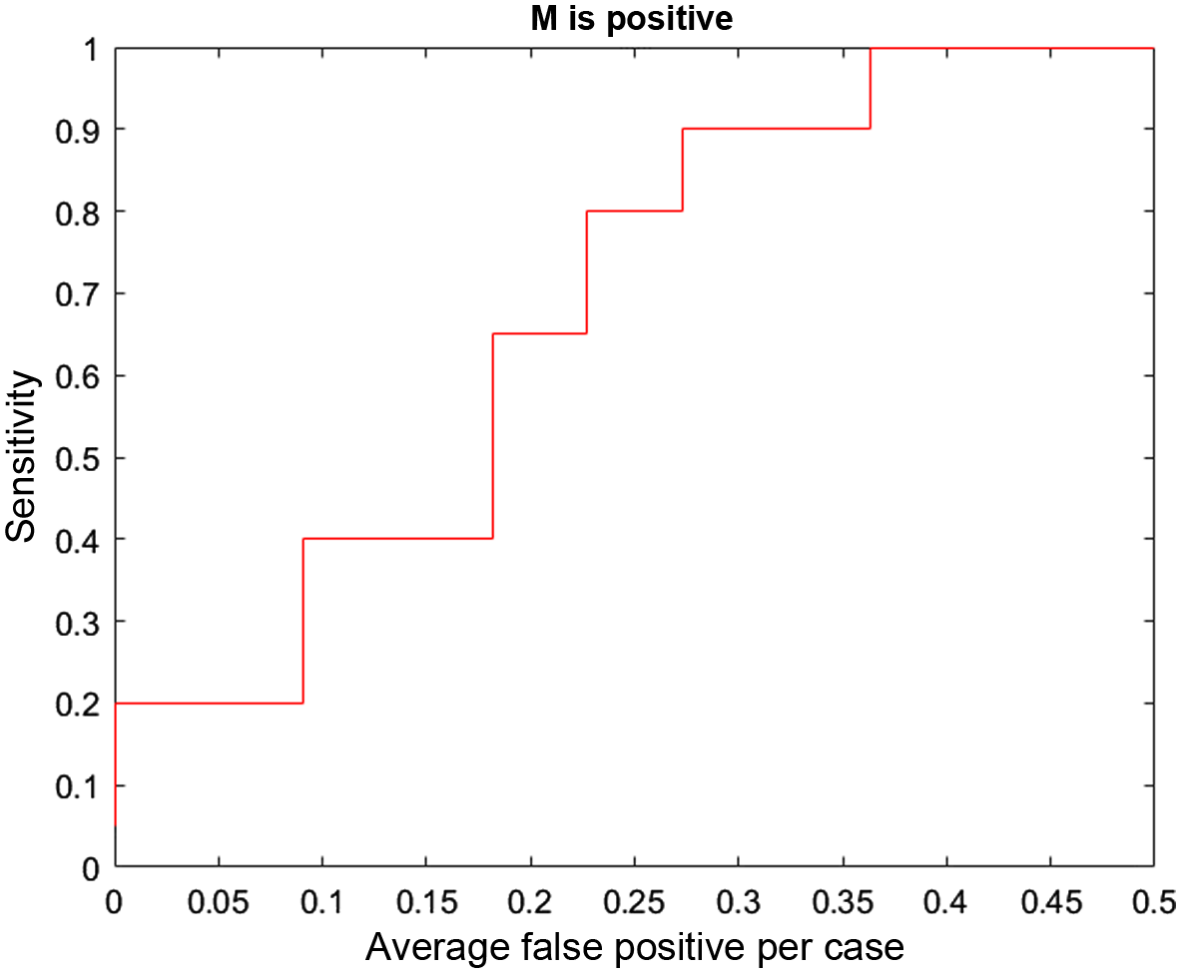
FROC curve based on proximity and correct classification as malignant.

The above sensitivity and average false positives per case would produce one point on an FROC curve. But there is an issue in producing other points on the curve. One of the ways that UDF has been implemented in other applications is to produce fused detections from the best hypothesis for the association of detections across the different data sources, and that is the approach used here. There is no threshold for a fused detection that can be varied to produce an FROC curve. Instead there is one best hypothesis. Another way that UDF has been implemented is to release fused detections according to a threshold on the probability of correct association (POCA) that is computed by UDF. In future implementations if a POCA threshold is used, then an FROC curve can be generated. In the meantime, there is another way to produce an FROC curve using the threshold on the probability of malignancy computed by UDF for joint classification. Another definition can be used that a fused detection is a true positive if the lesion is contained in the 95% error ellipse and is classified correctly as malignant. Then the following FROC curve is produced by varying the threshold for lesion classification (see Fig. 14).

The DBTex challenge is described in Ref. 25 where participants achieved >90% mean sensitivity at 1, 2, 3, and 4 FPs per volume. The authors state that they believe that one of the areas that should be focused on for future work is incorporating detection information from all available views (CC/MLO), mimicking the way radiologists analyze the images. In fact, that is one of the applications for which our UDF approach is designed. It is difficult to compare UDF performance directly with DBTex performance because UDF uses the type of ML approaches in DBTex as inputs to try and improve ML false alarm rejection. A direct comparison could be made if the ML algorithms of DBTex were used as input to UDF on the DBTex dataset. In the absence of performing that analysis, the approach described above to determine the ability of UDF to reject false alarms, perform joint classification, and produce 95% location error ellipses has been used.

The focus of the ML work for the UDF ML approach has been on the ML classification used by UDF to fuse data that are not only consistent in location parameters but also in classification decision variables. Improvements are now needed in ML detection to produce a higher percentage of true detections for UDF to realize its full potential. More sophisticated ML detection methods can be used. Also improvements can be made by lowering ML detection thresholds to improve the ML PD while still maintaining an overall acceptable UDF FAR as in other applications. This will lead to many more hypotheses in the UDF multiple hypothesis approach and a possibly excessive computational burden. But the use of multi-dimensional assignment methods based on Lagrangian relaxation has solved the computational problem in other applications.^26^ In addition, with improvements in nonlinear registration and location estimation mentioned previously, UDF performance is expected to improve. Also the PD can be improved by grouping similar fused detections for dim targets in other applications, and those grouping methods can be extended for this application.

## 4 Discussion

In this project, an artificial intelligence algorithm using UDF was developed to detect breast cancer. Multiple views of each lesion were used, combining DBT mammography CC and MLO views as well as US. The UDF algorithm has been applied to a set of test cases, with promising results. When ML made correct detections in all three views, UDF was able to create a correct fused detection in 90.9% of the cases. The UDF algorithm was able to process multiple separate detections from the CC and MLO mammographic views and breast US, integrating or “fusing” them to create one or two much smaller regions of interest. In many cases, the final fused region of interest contained the actual breast lesion, and the algorithm also correctly categorized the lesion as benign or malignant.

For the cases in the test set, ML processing of the images showed a maximum of five detections per image and most of the images had five detections. This would yield up to 15 detections per case (5 each for CC, MLO, and US images). After UDF processing, the number of detections was reduced to one or two fused detections, substantially reducing the number of false alarms for each case.

UDF has been used successfully in military target detection and characterization.^17^^,^^18^^,^^27^ Based on its success in other fields, fusing and interpreting two or more “views” of the same target, it is thought that UDF could be very useful for breast cancer detection. Breast imagers routinely obtain more than one view of potential target lesions, as standard mammograms contain both CC and MLO views. With tomosynthesis, there may be multiple slices through a lesion in both the CC and MLO views, adding to the views of the target lesion. US images contribute additional views and additional information about the same target lesion. UDF seeks to leverage the information from all views for detection and characterization of lesions. Previously described breast imaging AI algorithms have focused on detection and classification of lesions in a single view (CC or MLO views, single DBT slices, or US only).^12^ The results of other AI algorithms have been promising for breast cancer detection, characterization, and triage of cases based of probability of malignancy. The fusion techniques described here could contribute to existing AI algorithms. This project is a continuation of previously described work on UDF, presented at IWBI 2020 and 2022,^28^^–^^30^ as well as ongoing work on image registration.^24^

The main limitation of this work is the small number of cases evaluated. This study included only patients with single mass lesions. Other lesion types, such as calcifications, asymmetries, and architectural distortions, as well as studies with multiple lesions, were excluded. The overall performance of the UDF algorithm for cancer detection was hampered by ML detection limitations.

Future research would include processing a larger number of cases to assess the overall performance of the UDF algorithm for evaluation of combined mammography and US images. Most breast imaging patients undergo bilateral screening mammography, including CC and MLO views of each breast, with or without tomosynthesis. A focus on registration of lesions and fusion of detection and characterization information from the two standard mammographic views may provide a substantial improvement in performance over current efforts aimed at evaluation of one mammographic view at a time. In this study, UDF performed well if ML was able to correctly detect lesions in each view; therefore, future research should be directed toward improvement of ML detection, so that the UDF algorithm can reach its full potential.

## 5 Conclusion

An AI algorithm has been developed for detection and characterization of breast lesions, leveraging a combination of DBT mammography and US. ML, image registration, and UDF have been combined to yield promising results on a group of test cases.
